# Anti-androgenic effects of flavonols in prostate cancer

**DOI:** 10.3332/ecancer.2015.585

**Published:** 2015-10-22

**Authors:** Tristan Boam

**Affiliations:** Royal Liverpool University Hospital, Prescot Street, Liverpool, Merseyside, L7 8XP, UK

**Keywords:** androgen, chemoprevention, fisetin, flavonoids, flavonols, kaempferol, myricetin, prostate cancer, quercetin

## Abstract

Dietary-derived agents, such as the flavonoids, are of particular interest for prostate cancer (PCa) chemoprevention as they may offer a favourable safety and side-effect profile. An agent that demonstrates action on the androgen receptor (AR) axis may have value for preventing or treating castrate-resistant PCa. Four main flavonols – quercetin, myricetin, kaempferol, and fisetin – have been demonstrated in laboratory studies to have chemopreventive action in both castrate-resistant and castrate-sensitive PCa models. Mechanisms of flavonol action on the AR axis in PCa have been proposed to be inhibition of the 5α-reductase enzymes, direct androgen competition, suppression of the AR complex and transactivation by coregulators such as c-Jun, Sp1, and the PI3K/Akt pathway. It is, however, still unclear with current levels of evidence whether AR axis-mediated effects can fully account for the flavonols’ chemopreventive action.

## Introduction

Prostate cancer (PCa) is a leading cause of morbidity and mortality within the male population. It was the cancer with the highest incidence rate in men in the United Kingdom in 2013, a total of 40,372 new cases were diagnosed, accounting for 27% of all new male cancer diagnoses [1]. There is increasing evidence that a western diet along with other environmental influences may be a significant risk factor for the development of PCa. A lower incidence is observed in vegetarians and residents of Japan and China where diets are composed of more fruits, vegetables and soy products rather than processed and high glycaemic index foods [2, 3]. Interestingly, migrants from Asian countries eventually develop a PCa risk approaching that of their western counterparts, suggesting that diet and lifestyle factors have a significant role to play in the pathogenesis of PCa [4–9]. Recently, interest has turned towards identifying dietary components that may exert an anti-carcinogenic effect on PCa. Early phase-I and phase-II clinical trial results of dietary-derived agents, such as lycopene, curcumin, and isoflavone, show that they are well tolerated and have favourable effects on PCa activity markers, such as prostate-specific antigen (PSA) and serum testosterone levels [10, 11]; however, further evidence is needed to determine their value for the prevention or treatment of PCa.

**Figure 1.**
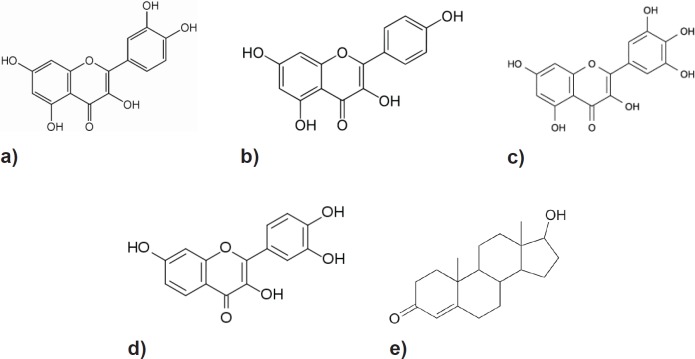
Structure of the four main flavonols: (a) Quercetin, (b) Kaempferol, (c) Myricetin, (d) Fisetin and (e) Testosterone.

The flavonols, a class of phenolic phytochemicals exemplified by quercetin, myricetin, kaempferol, and fisetin (Figure 1) can be found in foods, such as olives, onions, kale, and cranberries [12]. They have been shown to arrest PCa growth, invasion, and metastasis in laboratory studies *in vitro* and *in vivo* [13–20]. A US-led project investigating the potential use of fisetin to treat androgen-dependent PCa is currently in its pre-clinical stages. (US Appl No: 12/412945, 2009)

Their structural similarity to androgens (testosterone is shown) is evident, and hence, AR receptor interaction has been postulated as a mechanism for their anti-carcinogenic activity in PCa.

Development of PCa is thought to be a multistep process from the normal prostate epithelium to intraepithelial neoplasia (benign prostatic hyperplasia is not currently considered as a precursor to PCa), then localised carcinoma before progression to invasive or metastatic disease. Treatment options for advanced or metastatic disease are limited to androgen ablation and chemotherapy. Initially, most cancers are responsive to this; however, after a period of time, recurrences are common as the disease progresses to a castrate-resistant phenotype (CRPCa), for which there is a very poor prognosis [21]. Transition to CRPCa is likely due to changes within the androgen-receptor (AR) signalling axis and so this mechanism has become an attractive target for research into novel therapies [22].

ARs are present in most human tissues; however, they are most important for driving the development of sexual characteristics. The AR-signalling pathway is essential for maintaining normal metabolic function, cell proliferation, and homeostasis [23] and is an important component in the early pathogenesis of PCa. Regardless of whether prostate tissue is normal or neoplastic, its growth and survival is still dependant on AR signalling. The difference lies in the fact that CRPCa does not require an external stimulus of androgens. Many potential reasons for this exist, the most common stemming from AR mutations and aberrant post-translational modifications, causing the system to become constituently active even in the absence of external androgens. AR overexpression leading to hypersensitivity, activation by coregulators, non-androgenic ligands, and alternative pathways may also play a role [24].

The AR axis has previously been a target for chemopreventive agents in PCa. The 5α-reductase inhibitors finasteride [25, 26] and dutasteride [27] were the subject of clinical trials that seem to show a benefit of these agents for PCa chemoprevention. The systemic reduction in dihydrotestosterone (DHT) levels associated with an apparent reduction in PCa incidence in the trial participants may represent an AR axis-mediated role for chemoprevention. The 5α-reductase inhibitors, however, unfortunately have sexual side-effects and are currently still not licensed for PCa chemoprevention.

## Androgen competition

Flavonols are related structurally to oestrogens and this similarity has led to the hypothesis that they exert their action either by competing with androgens for AR binding sites or by affecting endogenous androgen levels.

Myricetin, quercetin, and fisetin have a catechol or pyrogallo configuration in the B-ring which may allow them to inhibit 5α-reductase isoenzyme 1, while kaempferol has been shown to inhibit isoenzyme 2 in transfected Rat 1A cells [28]. This may suggest that regular inclusion of these compounds in the diet may reduce DHT levels and consequently the androgenic stimulated growth of PCa. Kaempferol has also been shown to inhibit DHT stimulated cell growth in LNCaP cells and in contrast to the previous study, reduce expression of the 5α-reductase isoenzyme 1 gene [29]. This discrepancy may have arisen because of the difference in cell lines used but nevertheless supports the hypothesis that the flavonols have some activity on the 5α-reductases. It is still unclear, however, if a possible reduction in DHT levels would contribute a major androgen-dependant effect on their chemopreventive action.

Quercetin has been shown to compete with testosterone at high concentrations. However, the concentrations are higher than needed for cell growth inhibition, suggesting that this is unlikely to be its main cytostatic mechanism [30]. Another study found that quercetin increases serum testosterone, and after an initial rise, decreases DHT in a dose-dependent manner in a rat model. The rat prostates were found to have dilated lumens full of secretory materials. When coadministered with finasteride, it was shown to reduce rat prostate weight and negate the DHT-lowering effect of finasteride. The group hypothesised that quercetin acted synergistically with finasteride through an androgen-independent pathway to reduce the prostate weight. Quercetin may therefore hold value in combination to reduce the dose and therefore adverse effects of finasteride in benign prostatic hyperplasia or patients with PCa [31]. The results of these studies do not support quercetin as acting through androgen level modulation and displacement as its main chemopreventive action.

Khan *et al* observed that fisetin suppresses the growth of CWR22rv1 and LNCaP cells *in vitro* and competes with a labelled high-affinity androgen for the ligand-binding domain of the AR and in addition inhibits DHT-driven stabilisation of AR, leaving it vulnerable to proteasome degradation [32]. This would point to an AR-mediated effect being a large component of fisetin’s mechanism of action *in vitro*; however, there is a lack of other studies to support this hypothesis. It is also possible that fisetin was acting through a separate pathway, and the effects on the AR pathway are a consequence, rather than a cause of this action.

## Androgen receptor expression and activity

Many studies have focused on the effect of flavonols on the expression of the AR itself and its activity in the form of AR-dependant genes. AR luciferase reporter genes have been used to demonstrate that quercetin [33] and Kaempferol compounds [34] antagonise AR activity in the LNCaP cell line.

In terms of AR expression itself, quercetin seems to have varying effects. A reduction in AR expression in quercetin-treated CWR22Rv1 cells was observed in serum-free cultures, yet an increase in expression was seen when 10% foetal bovine serum was added [35]. In the LNCaP cell line, quercetin inhibited AR protein expression (36). Quercetin has also been shown to activate the mutant AR T877A (a variant able to respond to multiple ligands); however, in the same study, it increased overall AR expression while suppressing AR mRNA levels [37].

The AR-dependant genes PSA, 5α-reductase and human kallikrein 2 (KLK2) serve as markers of AR activity. Quercetin was shown to suppress the gene expression of PSA and KLK2 at the transcriptional level [36, 38], while kaempferol exhibited the same activity with the PSA and 5α-reductase genes, also interestingly inhibiting AR accumulation in the nucleus [29]. In one study, however, quercetin treatment was shown to increase PSA levels [37]. High PSA levels are associated with a worse prognosis in PCa and it is unclear whether a reduction in levels by flavonols would have a beneficial effect in itself or whether it serves as a marker of chemopreventive action. Khan *et al* were able to demonstrate a less ambiguous effect of fisetin in their study. In the CWR22rv1 and LNCaP cell lines, treatment with fisetin suppressed AR expression, inhibited protein and mRNA expression of AR target genes, and reduced PSA levels in a mouse xenograft (32).

These studies show that there is likely to be a suppressive effect of flavonols on the AR expression and activity. They are able to achieve this in the androgen-dependant LNCaP cell line and the more insensitive CW22Rv1 line. This may be a sign that flavonols have potential for the prevention or treatment of CRPCa via action on the AR axis, independent of direct ligand-receptor stimulation.

## Androgen receptor transactivators

Yuan *et al* have looked extensively into the activity of quercetin on AR transactivation by coregulators, hypothesising that these may the main mechanism of action of its effects on the AR rather than a direct interaction. An increased expression of the coregulator c-Jun was observed in LNCaP and LAPC4 PCa cells treated with quercetin. It is thought that c-Jun works to inhibit AR expression, and in this study, an inhibition of AR at the transcriptional level was observed [39]. Sp1 is another coregulator that has a promotional effect on the AR, quercetin was shown to attenuate this effect possibly by increasing Sp1-binding affinity for the C-terminal domain of the AR. Androgen-dependant post-translational phosphorylation of the AR was also reduced by quercetin [38], suggesting a blunting of its action. A later study suggested that quercetin achieves its anti-androgenic effect by promoting a c-Jun-Sp1-AR complex that reduced overall AR activity [40].

Aalinkeel *et al* were able to demonstrate that quercetin induces apoptosis in the largely androgen-independent PC-3 and DU145 PCa cell lines as a direct result of their inhibition of heat shock protein 90 [15], a key regulator of AR nuclear translocation [22, 41]. Whether this implies that an AR-mediated effect was contributory to the apoptotic process remains unclear.

The phosphatidylinositol 3-kinase/Akt (PI3K/Akt)-signalling axis serves as an AR transactivator, with Akt-dependant phosphorylation of the AR increasing its transcriptional activity [24, 42]. Flavonols have been shown in multiple studies to suppress Akt in conjunction with a growth-inhibitory effect on PCa cells [18, 19, 43–45]. A quercetin analogue-based PI3K inhibitor was developed as a PCa-specific prodrugactivated by PSA cleavage [46]. Inhibition of PI3K in this manner may suppress Akt-driven transactivation of AR, and hence, this drug may have a more potent anti-androgenic effect than unmodified quercetin.

## Conclusions

The hormone-refractory nature of advanced PCa has meant that the AR has been a target of much research, especially into dietary-derived potential chemopreventive agents, such as the flavonols. Quercetin has been the most extensively studied of this group, with evidence lacking as to the other compounds’ effect on the AR axis.

Whether or not flavonols exert their main effect through androgen competition remains ambiguous. All four main flavonols seem to have an inhibitory action on the 5α-reductases, hypothetically reducing DHT levels and blunting androgenic stimulation of growth in PCa. It is unclear, however, whether this would translate into a clinically relevant effect that is similar to finasteride and dutasteride. There is little evidence to show that flavonols directly compete with androgen binding to the AR. Fisetin demonstrated this ability in one study whilst quercetin was only able to compete at concentrations higher than needed to achieve its cytostatic effect. As the flavonols seem to exhibit an AR-mediated effect on the largely androgen-independent cell lines CW22Rv1 and PC-3 [14] without convincing evidence that they compete directly with androgens, it is reasonable to assume that they exert their action on the AR through a separate pathway.

While it is undeniable that these flavonols exhibit action on the AR, it remains unclear, and in some places unlikely that their main effect is AR axis mediated. Indeed, flavonols have shown efficacy in many cancer types, such as lung and colon, that are not reliant on androgenic stimuli for progression [47, 48]. It is possible that flavonols may act via AR transactivators. Quercetin, kaempferol and fisetin have all been shown to reduce expression of the AR itself as well as its transcriptional activity on AR-inducible genes. Yuan *et al* hypothesised that quercetin’s action was mediated through the co-regulators c-Jun and Sp1; however, it remains to be seen whether kaempferol and fisetin act in a similar way.

If the AR-signalling pathway is indeed the key to preventing or treating CRPCa, then methods to enhance flavonols’ effect on it may be beneficial. Structural modification of the flavonols, for example the quercetin-based PI3K inhibitor mentioned previously, is a potential avenue for increasing their action on the AR pathway. Another potential solution is using them in combination with other agents, for example other flavonoids [35, 45, 49, 50] or more well-established anti-androgenic agents, such as finasteride [31] and tamoxifen [51] which have demonstrated efficacy in tandem with quercetin in animal models of PCa.

In conclusion, flavonols exhibit a modest effect on the AR-signalling axis, but their value as chemopreventives or adjuncts to existing or novel treatments for CRPCa is promising.

## Conflict of interest

The author declares that they have no conflict of interest.

## List of abbreviations

PCa: Prostate cancer
CRPCa: Castrate-resistant prostate cancer
AR: Androgen receptor
DHT: Dihydrotestosterone
PSA: Prostate specific antigen
KLK2: Human kallikrein 2
